# Gut Microbiota and Metabolism in Different Stages of Life and Health

**DOI:** 10.3390/microorganisms10020474

**Published:** 2022-02-21

**Authors:** Shanthi G. Parkar, Pramod K. Gopal

**Affiliations:** 1 Seed Health Inc., Venice, Los Angeles, CA 90291, USA; 2 New Zealand Institute for Plant and Food Research, Palmerston North 4442, New Zealand; pramod.gopal@plantandfood.co.nz; 3 Riddet Institute, Massey University, Palmerston North 4442, New Zealand

In a very fascinating read, John Goodsir, a Scottish surgeon, describes how he isolated “vegetable organisms” from the “ejected fluid” from the stomach of his 19-year-old patient. He named the organism *Sarcina ventriculi* and prescribed antibacterial creosote drops, resulting in the elimination of symptoms [1]. This was probably the first case where human gastrointestinal microbiota was associated with disease. Since then, many more causative links have been established between microorganisms and infectious diseases such as typhoid fever, tuberculosis, and plague [2,3,4]. The advent of omics technologies has accelerated our understanding of the role of the gut microbiota in human physiology. Microbes are now known to modulate vitamin biosynthesis [5], maturation of the gastrointestinal tract and the immune system [6], protection against pathogens [7], and the development of non-communicable diseases such as obesity, metabolic syndrome, asthma and cancer [8].

Natural history studies of the human gut microbiome present the new-born infant to harbor a sparse gut microbiome [6,9], which then evolves by about 3 years of age to a 3 to 10 trillion cell community [6,10]. Healthy development of the gut microbiome within the first 1000 days of life is believed to be important for protection against many diseases later in life [11,12]. A diet incorporating fiber-rich or fermented foods has been shown to modulate gut microbiota in humans, from infants to adults [11,13,14,15]. The fiber reaches the large intestine and serves as a source of carbon for the microbial community, which in turn generate metabolites, mainly short chain fatty acids (SCFAs) that nourish both the microbiota and the host [7,16].

This Special Issue includes nine original articles and reviews that encompass the microbiome story at different stages of life and health. Three papers focus on the role of maternal microbiome or various foods on developing infant gut microbiome through weaning periods. In a review on the maternal microbiome, the authors examine how the mother’s oral, gut, and vaginal microbiota changes throughout pregnancy in parallel with her hormonal levels [17]. The review examines maternal microbial dysbiosis mediated mechanisms contributing to gestational diabetes and preeclampsia in the mother, and fetal growth restriction, preterm/still birth, or infections in the infant. Once the infant is born, the food ideally recommended is the mother’s milk, as it helps seed and feed up to 30% of the infant gut microbiota [18,19,20]. In a study based out of Indonesia, the authors followed the microbiome in 51 mother–infant dyads from birth to two years of age [21]. The dominant microbes in the mothers’ milk were *Staphylococcus* and *Streptococcus*, while maternal feces were dominated by *Prevotella* 9 and members of the *Clostridium* cluster IV. Feces of 3-month-old infants were dominated by *B. longum* subsp. *infantis* and *B. bifidum*, that are well-known for their capacity to metabolize the oligosaccharides in breastmilk [22,23]. As the infant grows, it is recommended that a more complex diet, including plant-based foods, meat and dairy, complement the breastmilk to meet the infant’s growth and development needs [11]. Thirty-two ingredients relevant for the formulation of infant-complementary foods were shown to differentially modulate the developing infant microbiome using an in vitro gut model [24]. The well-validated laboratory simulation of digestion and colonic fermentation employed in this study also offers an opportunity to examine how food blends can support age-specific or personalized changes in the developing infant microbiome [25,26].

Three original studies in this Special Issue describe health–microbiome interactions in three disease states. The study by Esparbès and colleagues examined the subgingival microbiota and cytokines in adult volunteers with periodontitis [27]. This is the first study comparing these parameters in healthy and diseased sites in the oral cavity of the same individual. The diseased oral sites had higher relative abundances of Synergistetes and Spirochaetes, while the healthy sites were enriched in Actinobacteria. They also identified *Desulfobulbus*, *Filifactor* and TM7 as potential biomarkers of periodontitis, concurring with data reviewed by Patini and colleagues [28]. The second study is a pilot clinical trial examining microbial differences at the species level between morbidly obese and normal weight subjects [29]. Using a commercially available qPCR-based Precision Microbiome Profiling (PMP™) method they found significant changes in the relative abundance of 17 of the 104 species analyzed. Of note were the decreases in *Akkermansia muciniphila*, two *Bifidobacterium* species, many butyrate producers including *Faecalibacterium prausnitzii* and *Ruminococcus bromii*, and one methanogen *Methanobacter smithii*. Many of these microorganisms have shown significant inverse correlations with body mass index in previous studies [30,31,32,33,34]. The third study is the first of its kind to detail the microbiome changes in adults with phenylketonuria (PKU) [35]. The key finding of this study was that, compared to the control group, PKU positive gut microbiome was poorer in the clostridial members *Faecalibacterium*, an unknown Lachnospiraceae and *Romboutsia*, and richer in *Enterocloster* (also a Lachnospiraceae member). Further metagenomic studies will provide useful insights to evaluate dietary strategies for PKU patients.

The role of the gut microbiota in dietary fiber metabolism to SCFAs and the consequent physiological outcomes is well characterized [7,11,16]. Microbiota also breaks down other diet-derived molecules such as polyphenols to generate metabolites that have a prebiotic effect [36,37]. Kiwifruit is a source of fiber and polyphenols that have been shown to be accessed and metabolized by gut microbiota, using an in vitro model of gastrointestinal digestion and fermentation [38]. Parkar and colleagues used a similar in vitro approach again to demonstrate that the digested green and gold-fleshed kiwifruit are rich in precursors of dopamine and serotonin, respectively [39]. At the colonic fermentation stage, the green and gold-fleshed kiwifruit increased L-dihydroxyphenylanine (L-DOPA, the dopamine precursor) and γ-aminobutyric acid (GABA), respectively. The digesta and fermenta, when incubated with gut epithelial cells, modulated genes related to gut tight junction, inflammation, and circadian rhythm. This study indicates that kiwifruit is potentially a source of physiologically relevant biogenic amines that are associated with functions such as sleep [40]. Indeed, kiwifruit has been shown to potentiate sleep via pathways implication serotonin and GABA [41,42]. On a similar theme, another article reviews edible mushrooms as microbiome-modulating functional foods [43]. Mushrooms are rich sources of β-glucan polysaccharides that have a prebiotic effect. The authors also review herbal beverages and their polyphenols, which act both as antioxidants and as prebiotics. Both mushrooms and herbal teas are also a source of microRNAs (miRNAs) that can interact with gut bacteria. 

Lastly, this Special Issue features a review of gut microbial pathways resisting colonization of the pathogen *Clostridiodes difficile* in the large intestine [44]. *C. difficile* infection (CDI) causes severe diarrhea and colitis, with significant morbidity and fatality rates. While antibiotics are used for CDI management, microbial pathways may potentiate colonization resistance against *C. difficile*. Microbial metabolites, such as secondary bile acids, SCFAs, antimicrobials, or competition for nutrients such as proline, that are required by the pathogen for proteolytic fermentation, offer directions to develop new therapeutics against this gut infection. 

Collectively, the original articles and reviews in this Special Issue present a blend of valuable data and insights adding to the growing body of knowledge in the field of diet–microbiome–host interactions. 
